# Paradise – not without its plagues: Overwhelming Blastomycosis pneumonia after visit to lakeside cottages in Northeastern Ontario

**DOI:** 10.1186/1471-2334-5-30

**Published:** 2005-05-04

**Authors:** Malvinder S Parmar

**Affiliations:** 1 Division of Clinical Sciences, Northern Ontario School of Medicine Laurentian & Lakehead Universities, Sudbury & Thunder Bay, Ontario, Canada; 2 Department of Medicine, University of Ottawa, Ottawa, Ontario, Canada

## Abstract

**Background:**

Visiting lakes and cottages is a common leisure activity during summer among most Canadians and paradise for some. Various leisure activities are involved during these visits, including cleaning and 'airing' the cottage after long-winters, activities at the lakes and dock building etc, exposing the Canadians to moist soil and decaying woods – a source of white or tan mould – *Blastomyces dermatitidis *that may cause a flu-like illness to severe pneumonia that often remains a diagnostic challenge and results in delay in diagnosis and appropriate treatment thereby increasing associated morbidity and mortality.

**Case Presentations:**

Five cases of overwhelming acute blastomycosis pneumonia are presented. Four of the five patients presented within few weeks of their visit to the cottages and surrounding lakes and all were initially treated as "community acquired pneumonia" that resulted in delay in diagnosis and poor outcome in the first patient. The first case, however, taught an important lesson that led to high-index of suspicion in the others with early diagnosis and improved outcomes. Interestingly, all patients were obese and had a shorter incubation period and severe clinical course. The possible mechanism for early and severe disease in association with obesity is speculated and literature is reviewed.

**Conclusion:**

High-index of suspicion is important in the early diagnosis and appropriate management acute blastomycosis pneumonia to improve associated morbidity and mortality.

## Background

During the few summer months in Canada visit to cottages and lakes is a frequent leisure activity. Access to a second home – camp, cabin, chalet or cottage, whatever we call it – has a strong cultural significance for Canadians. The cottage, for many Canadians, is a paradise where extended family and friends gather together, where there is time for leisure and contact with nature.

Pulmonary blastomycosis can be difficult to diagnose and only 18% of patients were correctly suspected to have blastomycosis in an endemic area and are often initially misdiagnosed and treated as community acquired pneumonia, malignant tumor or tuberculosis resulting in unnecessary surgery and treatment delays[1]. Experience with five cases of blastomycosis pneumonia from Northeastern Ontario is presented emphasizing the high-index of suspicion for early diagnosis and improving outcome. The patients presented with overwhelming acute blastomycosis pneumonia within few weeks after visiting lakes and cottages. These were initially treated as community acquired pneumonia and a lesson learnt from first case is emphasized and a high-index of suspicion by the author led to early diagnosis in others. Interestingly all patients were obese and had severe disease. A possible association with obesity and severity of disease is observed and mechanism speculated.

## Case presentations

The demographics, month of presentation, time to illness and diagnosis after exposure and the outcomes are summarized in table 1. A brief history on all with a detailed history of case 3 is presented.

**Table 1:** A summary of 5 cases

Month of presentation to hospital	Age (years)	Gender	Weight (Pounds)	# of days symptoms started after visit to cottage or lake	# of days to diagnosis after onset of symptoms/after hospitalization	Outcome
July	30	Male	340	7–10	40/22	Died
July	34	Female	270	7	20/7	Recovered
July	36	Female	200	10	36/12	Recovered
July	36	Male	230	6	19/1	Recovered
February	57	Female	>350	Not clear	30/3	Died

### Case 1

In July 1998, a 30-year old man with morbid obesity [weight 340 pounds] presented to emergency department with 3-weeks history of 'flu-like' symptoms with fever, chills, night sweats and cough with yellowish phlegm that started after building a dock at his cottage. Chest x-ray revealed bibasilar infiltrates. He was admitted with bilateral pneumonia and was started on intravenous penicillin and oral clarithromycin after obtaining blood and sputum cultures. Three days later he developed rash and penicillin was discontinued and antibiotics were changed to intravenous ceftriaxone and erythromycin. He remained febrile for next 7 days. Sputum Gram stain showed many pus cells but cultures remained negative. His condition continued to deteriorate. Local bronchoscopist was unavailable to perform bronchoscopy and arrangements were made to transfer him to another hospital. Before transfer, his condition deteriorated and was intubated. Tracheal secretions were aspirated and sent for cultures – including fungal and tubercular. The night of transfer, he developed cardio-respiratory arrest and couldn't be resuscitated. Next day, the wet preparation on the tracheal secretions revealed thick walled budding yeast consistent with blastomycosis. Autopsy showed severe pulmonary disease with solidification of both lungs and cultures confirmed the diagnosis.

### Case 2

In July 2000, a 34-year old obese woman [270 pounds] presented to the emergency department with pleuritic chest pain associated with chills and night sweats about a week after she was camping at the lakeside cottage. She denied symptoms of cough or phlegm. She was diagnosed with musculoskeletal pain and discharged home on analgesics. Five days later she presented with ongoing symptoms of fever, chills with dry cough and right lower chest pain. A chest X-ray revealed an infiltrate in the right lower lobe. She was diagnosed with community acquired right lower lobe pneumonia and sent home on oral clarithromycin. She presented 4-days later with ongoing symptoms to the emergency department and the chest x-ray now showed worsening pneumonia. She was admitted to the hospital and started on intravenous ampicillin and ceftriaxone, and a ventilation-perfusion scan showed a matched perfusion defect in right lower lobe. She continued to feel weak with night sweats and chest X-ray showed worsening of infiltrate. A medical consult was requested. At this time, during consultation it was noted that her symptoms started after a short stay at the cottage that reminded me of the previous case, and the possibility of acute blastomycosis pneumonia was raised. Her white blood cell count remained slightly elevated at 13.0 but erythrocyte sedimentation rate was markedly elevated at 112. As she didn't have productive cough, a bronchoscopy was recommended that couldn't be performed locally [local bronchoscopist was away] and she was referred to a tertiary care center where bronchio-aleveolar lavage showed budding yeast and cultures confirmed growth of blastomycosis. She was treated initially with intravenous amphotericin B and later switched to oral itraconazole for a year and made full recovery.

### Case 3

**Figure 1:**
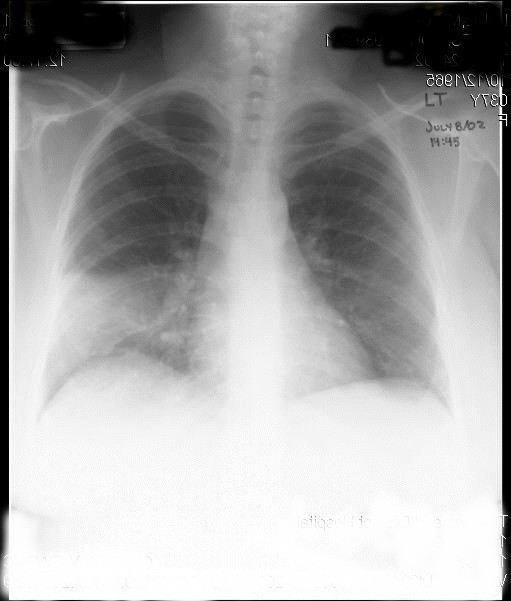
Chest X-ray (PA view) at initial presentation showing early right lower lobe consolidation.

In July of 2002, a 36-year old obese woman [weight 200 pounds, height 5 feet 1 inch] with history of type 2 diabetes for 5 years, presented to the emergency department with 5 days history of fever, chills and cough with yellowish phlegm and sharp pain in her right lower chest that aggravated with deep breathing. Physical examination was unremarkable. She was afebrile and lungs were clear. Homen's sign was negative. Initial laboratory data showed slightly elevated d-dimer of 0.374 ug/mL [normal <0.25 ug/mL]. A chest X-ray (figure 1) showed right lower lobe infiltrate. She was diagnosed with community acquired pneumonia and sent home on oral clarithromycin 500 mg BID, cefurox 500 mg BID for 7 days and acetaminophen as required.

**Figure 2:**
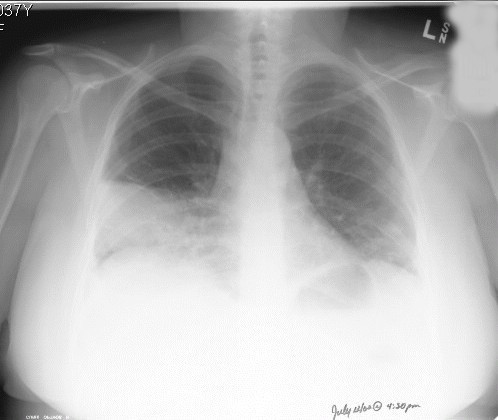
Chest X-ray (PA view), 5 days later, showing progression of disease with infiltrates in right and left lower lobes.

She continued to have symptoms of fever, chills, night sweats with productive cough and dyspnea and presented to the hospital five days later. She denied hemoptysis. Physical examination revealed a temperature of 39 degree Celsius, mild tachycardia with heart rate of 104 beats per minute, normal blood pressure of 125/74 mmHg and respiratory rate of 18. Pulse oxymetry revealed oxygen saturation of 96% on room air. There was decreased air entry with bronchial breath sounds in right lower chest. A white cell count was slightly elevated at 13.5, with normal hemoglobin of 128 g/L and d-dimer between 0.25–0.50 ug/mL. A second chest X-ray (figure 2) showed dense infiltrate in right lower lobe. She was admitted with the diagnosis of community-acquired pneumonia and started on intravenous levofloxacin 500 mg a day.

She had a positive Mantoux test in 1976. There was no history of travel save for visiting her sister's cottage at the local lake a weak before where she slept at the gazebo for two nights. There were no pets at home. She smoked one pack per day and entertained social drinks.

**Figure 3:**
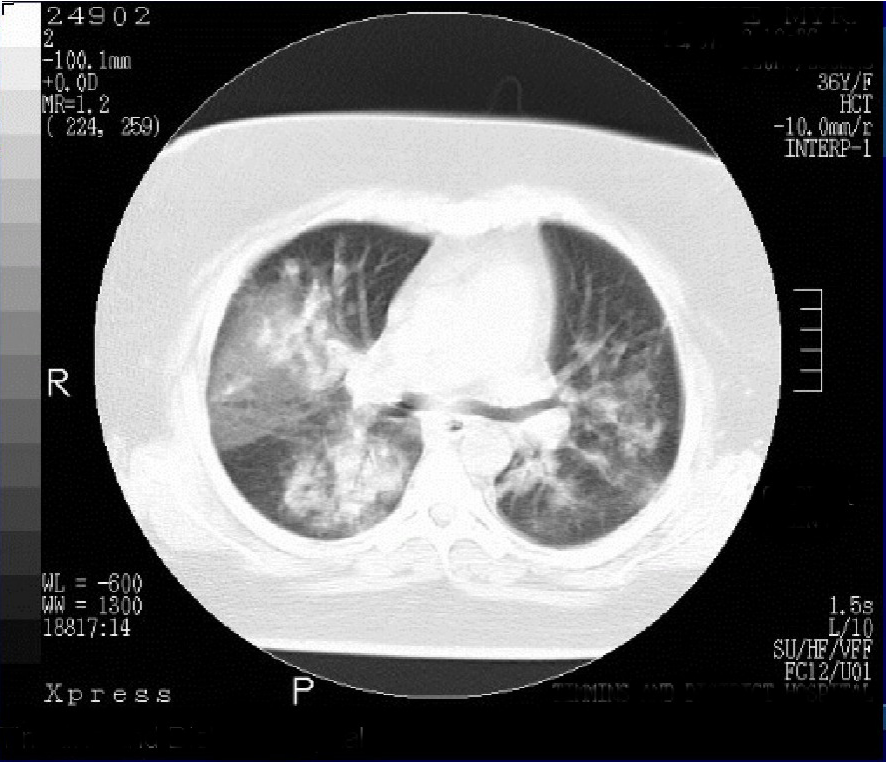
A section of CT scan of chest performed 8 days after hospitalization showing bilateral consolidation of lungs, mainly of lower lung fields.

She continued to have temperature up to 39 degrees Celsius, productive cough and shortness of breath. Sputum Gram stain showed 4+ neutrophils and 1+ normal flora and blood cultures remained negative. She received intravenous levofloxacin and clindamycin for one week. Bronchoscopy performed on day 6 was unremarkable save for inflammatory changes in lower lungs and samples were collected for bacterial and tuberculosis cultures [in retrospect, the specimen was not sent for fungal cultures]. On 7^th ^day because of ongoing symptoms, the antibiotics were changed to intravenous imipenem-cilastatin sodium 1 gm every 12 hour and she has had a CT scan of chest (figure 3) that showed bibasilar consolidation with sparing of the apices. The white cell count fluctuated between 13.6 to 20.2 [normal 4.0–11.0 × 10^9 ^/L]. Hemoglobin decreased to 97 g/L. BUN, serum creatinine, electrolytes, AST, ALT, GGT and ALP were normal. Urinalysis was negative. Blood, urine and sputum cultures remained negative. Sputum gram stain showed 3-4+ neutrophils without organisms. ESR on day 8 was elevated at 112 mm/hr [normal 0–15]. Antinuclear antibody was negative. Arterial blood gas on day 8 showed pH of 7.46, pCO_2 _of 35, pO_2 _of 59 and oxygen saturation of 91% on room air. She was started on supplemental oxygen by nasal prongs. Bronchoscopic specimen cultures were negative save for pending TB cultures.

After five days of intravenous imipenem-cilastatin sodium therapy, there was no improvement in her clinical condition. Another medical consult was requested, when the author got involved in her care. She had a temperature of 39 C, pulse rate of 104 and blood pressure of 114/70 mmHg. There was no lymphadenopathy and heart sounds were normal. Her chest examination showed decreased breath sounds at both bases with egophony in right lower chest. There were no pleural rubs. There was no clubbing or cyanosis. The rest of the examination was unremarkable save for an obese benign abdomen.

**Figure 4:**
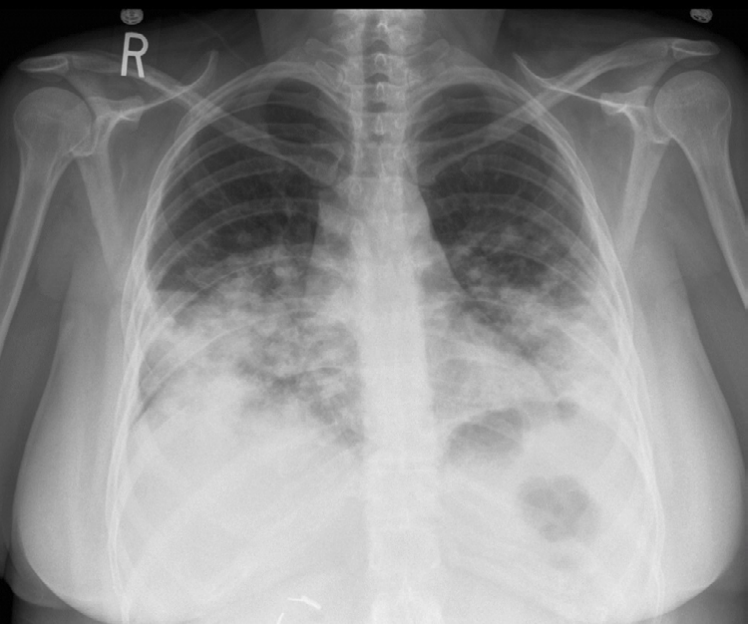
Chest X-ray (PA view) – after 8 days of intravenous antibiotic therapy, showing further worsening of bilateral lower lung disease with nodular pattern – raising suspicion of metastatic disease.

Chest X-ray (figure 4) now showed further deterioration of the bilateral infiltrates with nodular appearance and the radiologist remarked, "The lesions are suspicious of metastases." Sputum cytology showed a large number of acute inflammatory cells without malignant cells. The possibility of vasculitis was entertained because of markedly elevated ESR and antineutrophilic cytoplasmic antibody (ANCA) was negative.

**Figure 5:**
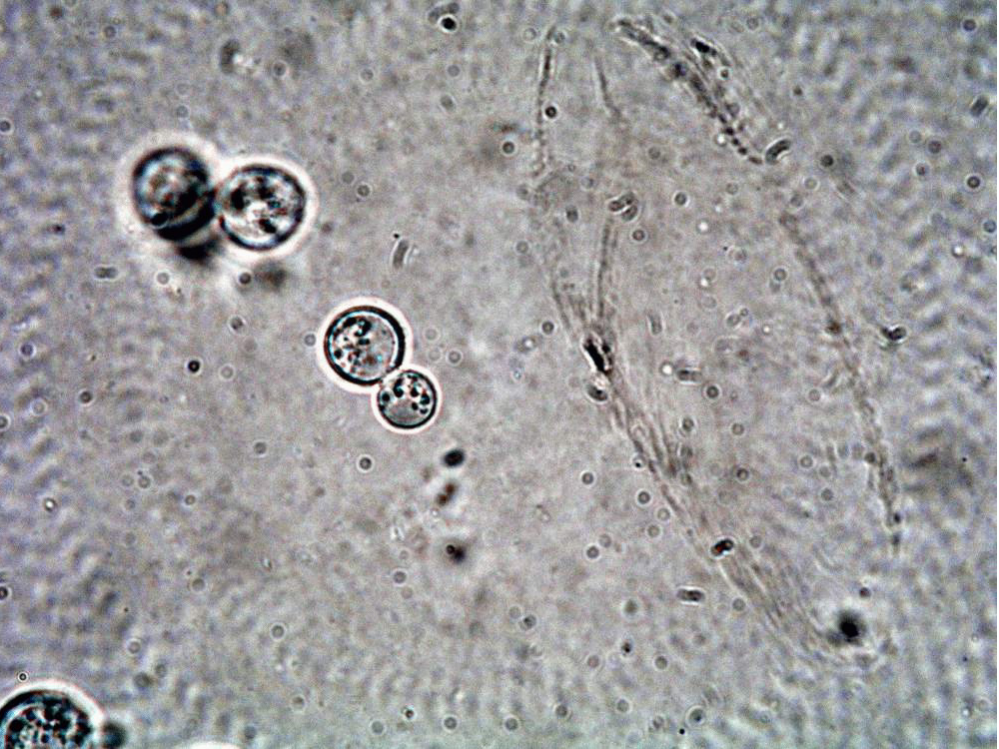
Wet preparation of sputum [25% NaOH with 5% Glycerol as the mounting medium, 40× magnification] showing budding yeast [blastocyst]

The history of sleeping at the cottage for two nights, a week before her illness started; progressive bilateral pneumonia with ongoing high-grade fever, chills, and night sweats despite sufficient antibiotic coverage and sputum Gram stain showing a large number of neutrophils without organisms (culture negative) raised the author's suspicion for the possibility of fungal process such as acute blastomycosis pneumonia and the Public Health Laboratory (PHL) was specifically asked to perform wet preparation for blastomycosis on the sputum. The PHL laboratory confirmed the presence of 'round thick walled budding yeast like cells (figure 5) suggestive of *Blastomyces dermatitidis *and this was confirmed on fungal cultures. She was promptly started on intravenous amphotericin B and responded well and later switched to oral itraconazole 400 mg twice a day for 6 months and then 200 mg a day for another 6 months. She recovered fully.

### Case 4

Same July of 2002, while case #3 was still-in hospital, a 42-year old man [weight 230 pounds] presented to a peripheral community hospital with symptoms of fever, chills and malaise about 7–10 days after a trip to the local beach. He was diagnosed with community acquired left lower lobe pneumonia and treated with three different kind of antibiotics. He remained febrile with temperature of 39–40 degree Celsius and 10-days after his admission, he was transferred to our hospital and again the past experience and a recent case of similar nature, although from a different region, reminded of the similar process and he underwent bronchoscopy and the wet preparation showed budding yeast and cultures were confirmatory. He was promptly started on intravenous amphotericin B and later switched to oral itraconazole and made full recovery.

### Case 5

**Figure 6:**
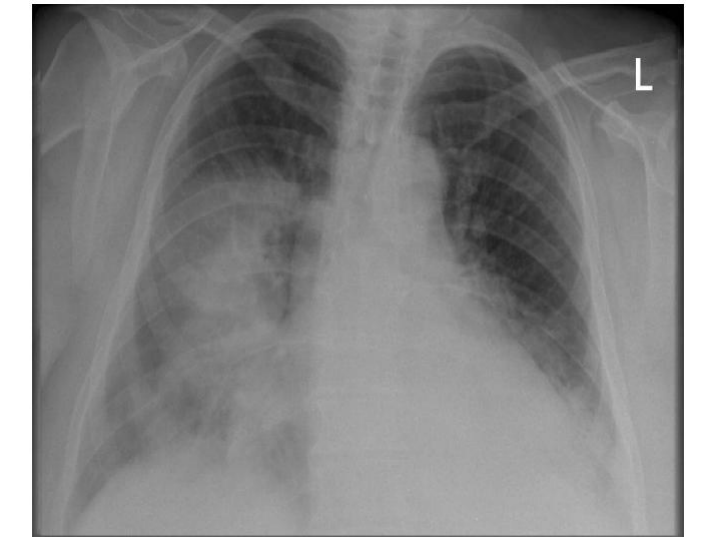
Chest X-ray (PA view) at initial presentation showing consolidation within the right mid and both lower lobes.

In February of 2004, a 57-year old woman with morbid obesity [weight over 350 pounds] with history of hypertension and type 2 diabetes presented with one month history of fever, night sweats and cough and was admitted to the hospital with right sided community acquired pneumonia [figure 6] and started on intravenous levaquin. She had history of gall stones and had mild upper abdominal pain. Next morning, while undergoing an ultrasound examination she became unresponsive and suffered cardio-respiratory arrest. She was successfully resuscitated, but remained hypotensive and required inotropic support and admitted to intensive care unit under my care. Because of shock and dense lobar pneumonia, the possibility of severe pneumococcal pneumonia was entertained and intravenous penicillin was added. She had copious amount of thick pus from the endotracheal tube and because of thick pus, possibility of acute blastomycosis pneumonia was entertained although it was -36 degree Celsius outside, and specimen was sent to Public Health Laboratory to perform a wet preparation to confirm or rule out the possibility blastomycosis. Although blastomycosis was suspected but empiric therapy with amphotericin B was not started, as there was no clear cut history of exposure and she presented in the middle of winters when the outside temperature was -30 to -40 degrees Celsius. She was difficult to ventilate and remained unstable during the night. Next morning she arrested and could not be resuscitated after 2-hours of resuscitation. At noon on that day, Public Health Laboratory confirmed the diagnosis of acute blastomycosis pneumonia.

## Discussion

Blastomycosis is a relatively rare but important and lethal disease and is caused by a thermal dimorphic fungus – *Blastomyces dermatitidis *– that exists in mycelial form in the environment and develops into yeast form in the host [2-4]. Clinical spectrum is variable and range from asymptomatic infection to pyogranulomatous inflammation with fulminant hypoxic respiratory failure or acquired respiratory distress syndrome (ARDS) to extrapulmonary manifestations[5,6]. Often acute pulmonary disease mimics a bacterial pneumonia or cause respiratory distress syndrome and pose a diagnostic challenge and results in delay in diagnosis, incorrect treatment and poor outcome.[1,5,7]

**Figure 7:**
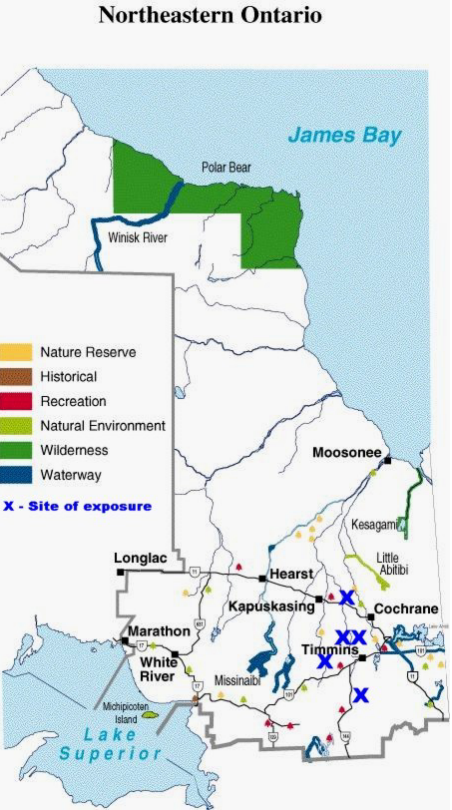
Map of Northeastern Ontario, showing the location of exposure [adapted from , accessed April 2, 2005].© Queens Printer for Ontario, 2002. Adapted and reproduced with permission

The incidence of blastomycosis remains largely unknown as it is not a reportable disease in Canada and was removed from the list of reportable diseases in Ontario in 1990[8]. It is an uncommon, though regularly seen, life threatening disease in endemic areas. In North America the disease is concentrated along the Mississippi and Ohio River basins, but also extends into Northern Wisconsin, Minnesota and the Canadian provinces bordering the Great Lakes[2,5]. In Ontario, it is endemic especially in Northwestern Ontario, north and west of Lakes Superior and Huron and in neighboring boreal Manitoba[9]. In the endemic areas the disease is predominantly encountered in rural or wilderness areas where the causative agent likely colonizes the soil or plant litter in riparian sites[10]. Outbreak studies have implicated, building of a hunting lodge, proximity to a construction site, raccoon hunting, exposure to a beaver lodge, and activities by riverbanks as sources of exposure. [9,11-13] A retrospective review from 1990 to 1998 in Northwestern Ontario revealed an incidence of 117 per 100,000 population with much higher rate of 404 per 100,000 population in aboriginals[14]. Public Health Laboratory in Timmins covers most of the Northeastern Ontario and identifies an average of 5–6 isolates per year [personal communication, PHL, Timmins]. Three of the patients [#1, 2 and 5] were from Timmins and one [#3] about 200 Km south and the other [#4] about 100 km North of Timmins (figure 7).

Four of the five patients presented in July compared to the reported occurrence between September and January. The incubation period was much shorter than the reported period of 21 to 106 days with an average of 45 days[10]. The reason for short incubation period is unclear but it is interesting that all patients were significantly obese and two had diabetes. Diabetes increases the risk for fungal infections and may be present in 22% of patients[1]. Whether obesity altered the course and severity of disease requires further study but it is known that obesity can alter pulmonary function by its adverse effects on respiratory mechanics, resistance within the respiratory system, respiratory muscle function and lung volumes[15] and thereby might cause decreased clearance of inhaled spores resulting in severe disease with shorter incubation period. Baik et al noted a two-fold increase in the risk of community acquired pneumonia both in men and women who gained over 40 pounds from their adulthood weight and a direct association with increasing BMI in women[16]. Obesity is also shown to impair T and B cell function [17,18] that may be mediated in part by consequences of obesity, such as hyperglycemia and insulin resistance[19]. It is possible that non-obese people might have had such exposure and were either able to clear the inhaled spores or developed a milder form of disease indistinct from flu-like illness.

### Laboratory diagnosis

The role of sputum culture in the management of community-acquired pneumonia is controversial and only 14.4% of patients yielded good-quality sputum in a recent study[20] and is not routinely recommended[21]. However, the yield from respiratory secretions [wet preparation and cultures] is high [80–100%] but is underutilized.[22] Wet preparation [10% potassium hydroxide] examination of respiratory secretions showing budding yeast is the gold standard initial test and cultures are confirmatory but may take up to 5 weeks. The first case taught an important lesson and re-enforced the old dictum – "*presence of a large number of neutrophils on the Gram stain indicates that the sputum is of good quality"*. When no organisms are seen or cultured from this quality specimen, it should raise the suspicion for non-bacterial (fungal, mycobacterial, etc.) causes. This lesson was helpful, especially to me, in making a timely diagnosis in other four patients, where the patients were treated initially with different antibiotics for "pneumonia" before my involvement. The diagnosis was suspected at my initial encounters and confirmed within 24–72 hours in all.

*Lesson: Sputum full of neutrophils indicates 'a good quality specimen' and with negative cultures should alert the physician to look for non-bacterial causes.*

### Radiographic features

The radiographic features of blastomycosis are highly variable and no one pattern exists[23], making differential diagnosis from other bacterial, fungal and neoplastic disease difficult. Often initial radiographic presentation tends to be localized airspace disease characterized by patchy and confluent airspace opacities with indistinct borders, in a segmental, subsegmental, or non-segmental distribution[3]. Due to these radiographic features and clinical presentation of fever, chills and productive cough, the initial diagnosis of blastomycosis is often overlooked in favor of a diagnosis of bacterial pneumonia. In chronic form, when presents as a focal mass it mimics bronchogenic carcinoma prompting additional tests such as needle biopsy and even a lobectomy[1,5].

### Treatment

Treatment of blastomycosis depends on the clinical presentation and presence or absence of extra-pulmonary manifestations. Patients with mild pulmonary disease whose symptoms are resolving at time of diagnosis require observation. All other patients with symptomatic pulmonary disease or extrapulmonary disease require antifungal therapy. amphotericin B is recommended for patients with severe acute pulmonary disease [as in the cases presented], with associated ARDS[5], immunocompromised hosts and in those with central nervous system involvement. Oral azoles – Itraconazole or fluconazole are recommended for mild to moderate acute, subacute or chronic pulmonary and disseminated forms and after intravenous amphotericin B therapy in severe cases. A 6 to12 months of therapy with antifungal agents is often required.

### Prognosis

The prognosis for most patients with mild to moderate pulmonary disease with or without dissemination is good provided that antifungal therapy is initiated promptly after diagnosis[23]. Mortality is about 10% in patients with severe pulmonary disease without ARDS but reaches close to 90% in patients with associated ARDS.[24]

### Prevention

There are no specific prevention strategies. However, activities that bring individuals closer to rotting wood or moist soil near water are associated with a greater risk and a high-index of suspicion especially in endemic areas with early and appropriate testing is important. Soil testing, even in endemic areas, is neither cost-effective nor reliable and is not recommended.

## Conclusion

Visit to cottages and lakes, is a paradise for most Canadians where they spend most of their summers relaxing and participating in outdoor activities that may expose them to *Blastomyces dermatitidis*. The five cases presented here are not to illustrate the drawbacks of these activities but to increase the awareness of blastomycosis to the public, visitors to endemic areas and healthcare providers. It is important for the patients to provide their history of stay at the cottages and lakes and contact with wet soil and decaying woods and for the physicians to be vigilant and consider this diagnosis early and request appropriate testing to reduce associated morbidity and mortality.

## Competing interests

The author(s) declare that they have no competing interests.

## Pre-publication history

The pre-publication history for this paper can be accessed here:


